# Pathogenic and therapeutic role for NRF2 signaling in ultraviolet light–induced skin pigmentation

**DOI:** 10.1172/jci.insight.139342

**Published:** 2020-10-15

**Authors:** Michelle L. Kerns, Robert J. Miller, Momina Mazhar, Angel S. Byrd, Nathan K. Archer, Bret L. Pinkser, Lance Lew, Carly A. Dillen, Ruizhi Wang, Lloyd S. Miller, Anna L. Chien, Sewon Kang

**Affiliations:** Department of Dermatology, Johns Hopkins University School of Medicine, Baltimore, Maryland, USA.

**Keywords:** Dermatology, Cellular immune response

## Abstract

Mottled skin pigmentation and solar lentigines from chronic photodamage with aging involve complex interactions between keratinocytes and melanocytes. However, the precise signaling mechanisms that could serve as therapeutic targets are unclear. Herein, we report that expression of nuclear factor erythroid 2–related factor 2 (NRF2), which regulates reduction-oxidation reactions, is altered in solar lentigines and photodamaged skin. Moreover, mottled skin pigmentation in humans could be treated with topical application of the NRF2 inducer sulforaphane (SF). Similarly, UV light–induced pigmentation of WT mouse ear skin could be treated or prevented with SF treatment. Conversely, SF treatment was unable to reduce UV-induced ear skin pigmentation in mice deficient in NRF2 or in mice with keratinocyte-specific conditional deletion of IL-6Rα. Taken together, NRF2 and IL-6Rα signaling are involved in the pathogenesis of UV-induced skin pigmentation, and specific enhancement of NRF2 signaling could represent a potential therapeutic target.

## Abstract 2

Pharmacological activation of NRF2-signaling resulted in improved skin hyperpigmentation in human and mouse skin.

## Introduction

Photoaging is the premature aging of skin as a consequence of chronic cutaneous exposure to UV radiation (UVR), most commonly from long-term sun exposure (1). Photoaging manifests clinically with wrinkles, loss of elasticity, and mottled hyperpigmentation (2). There has been a strong interest in the prevention and treatment of photoaging, as evidenced by the increasing number of over-the-counter and prescription products aimed at treating the clinical features of photoaging (3). Moreover, information regarding the UV radiation (UVR) response of keratinocytes and melanocytes may also have implications for other pigmentation disorders and skin carcinogenesis.

Solar lentigines are discrete hyperpigmented lesions characteristic of photoaging that often affect sun-exposed skin (4). They are also associated with an increased risk of skin cancer (5). Histologically, solar lentigines are characterized by club-shaped rete ridges, with small extensions and pigmentation restricted to the basal layer. The molecular mechanism of the initiation of solar lentigines is unclear, but it is thought to be due to abnormal melanogenesis and keratinocyte proliferation.

Skin pigmentation results from crosstalk between melanin-producing melanocytes and melanin-receiving keratinocytes. Melanocytes synthesize 2 types of melanin, eumelanin (black-brown) and pheomelanin (red-yellow), from a common precursor L-tyrosine and with the aid of tyrosinase. The ratio between these 2 determines skin color. One melanocyte supplies approximately 36 keratinocytes through arborization of dendritic processes, giving rise to an epidermal melanin unit (6). Newly produced melanin is transported in melanosomes along dendritic projections of melanocytes and transferred to keratinocytes in a process akin to phagocytosis (7, 8). Within keratinocytes, imported melanosomes fuse with lysosomes. Cytoskeletal elements and motor proteins then mediate the translocation of free melanin to the apical pole where they can absorb UVR. The size and distribution of melanosomes within the keratinocyte vary depending on skin color (9). As keratinocytes undergo terminal differentiation, the imported melanin is degraded through an unknown mechanism (10). Keratinocytes can also regulate melanocyte functions, including proliferation, differentiation, melanogenesis, and dendritogenesis, via direct cell-to-cell contact and the secretion of growth factors (11, 12). Keratinocytes have been shown to influence the pheomelanin/eumelanin ratio in cultured human melanocytes (13), and there is strong genetic evidence from both human diseases and mouse models that keratins can impact skin tone (14).

Although hyperpigmentation plays an important role in the skin’s protective response to solar UVR and other environmental stressors, it is also an indicator of and inextricably linked with skin damage. The exposure of the skin to both UVB (280–320 nm) and UVA (320–400 nm) irradiation results in the formation of ROS, which stimulate melanin synthesis but also lead to damage of nucleic acids, lipids and proteins (15). The skin has the capacity to mount a robust antioxidant response to combat the deleterious effects of UVR. Nuclear factor erythroid 2–related factor 2 (NRF2) signaling orchestrates transcriptional programs that facilitate the adaption and survival of cells in settings of oxidative stress. Under normal conditions, NRF2 is constitutively expressed in all skin cell types, and its activity is regulated by protein turnover via Kelch-like ECH-associated protein 1–mediated (KEAP1-mediated) ubiquitination and proteosomal degradation (16). Reactive cysteines in KEAP1 effectively serve as cellular reduction-oxidation (redox) sensors, and their modification by ROS and electrophiles lead to a conformational change, allowing the release, stabilization, and nuclear translocation of NRF2 (17). The autophagy receptor p62 can also interact with the NRF2-binding site on KEAP1, competing with the interaction between NRF2 and KEAP1. This results in stabilization of NRF2 and transcriptional activation of NRF2 target genes, which are instrumental for antioxidant, antiinflammatory, and detoxification cellular defense responses (18). Thus, the NRF2/KEAP1 redox sensitive signaling axis is primed to react rapidly to electrophilic and oxidative cellular stresses, including chronic UVA exposure and subphysiological doses of UVB (19, 20).

Progressive derangement of NRF2 signaling has been hypothesized to be a critical factor driving the age-related decline in antioxidant defense of several organ systems, including skin (21). The photoprotective effects of pharmacological activation of NRF2 have been demonstrated in cultured human cells and reconstructed human skin and mouse skin (22–24). With respect to skin pigmentation, alterations in NRF2 signaling have been observed in stress-induced loss of pigmentation disorders of both the skin and hair follicles, such as vitiligo and hair graying, respectively (25). Notably, the complex crosstalk between keratinocytes and melanocytes that mediates UVB-induced pigmentation has been shown to be affected by NRF2 signaling via an ROS-dependent mechanism in cultured cells (26). Taken together, alterations in NRF2 signaling have been implicated in aging and stress-induced skin pigmentation disorders in the skin and hair follicles, but the specific mechanisms involved are incompletely understood. Therefore, we set out to determine whether NRF2 was involved in the pathogenesis of solar lentigines that occurs in photodamaged skin and whether topical application of an NRF2 inducer sulforaphane (SF) could reduce skin hyperpigmentation in humans. Additionally, we used a mouse model of UV-induced hyperpigmentation to determine the mechanistic effects of NRF2 in skin hyperpigmentation and the potential for SF as a therapeutic intervention

## Results

### NRF2 and HO-1 had reduced expression in lentigines and older photodamaged skin compared with younger photodamaged skin.

**Figure 1:**
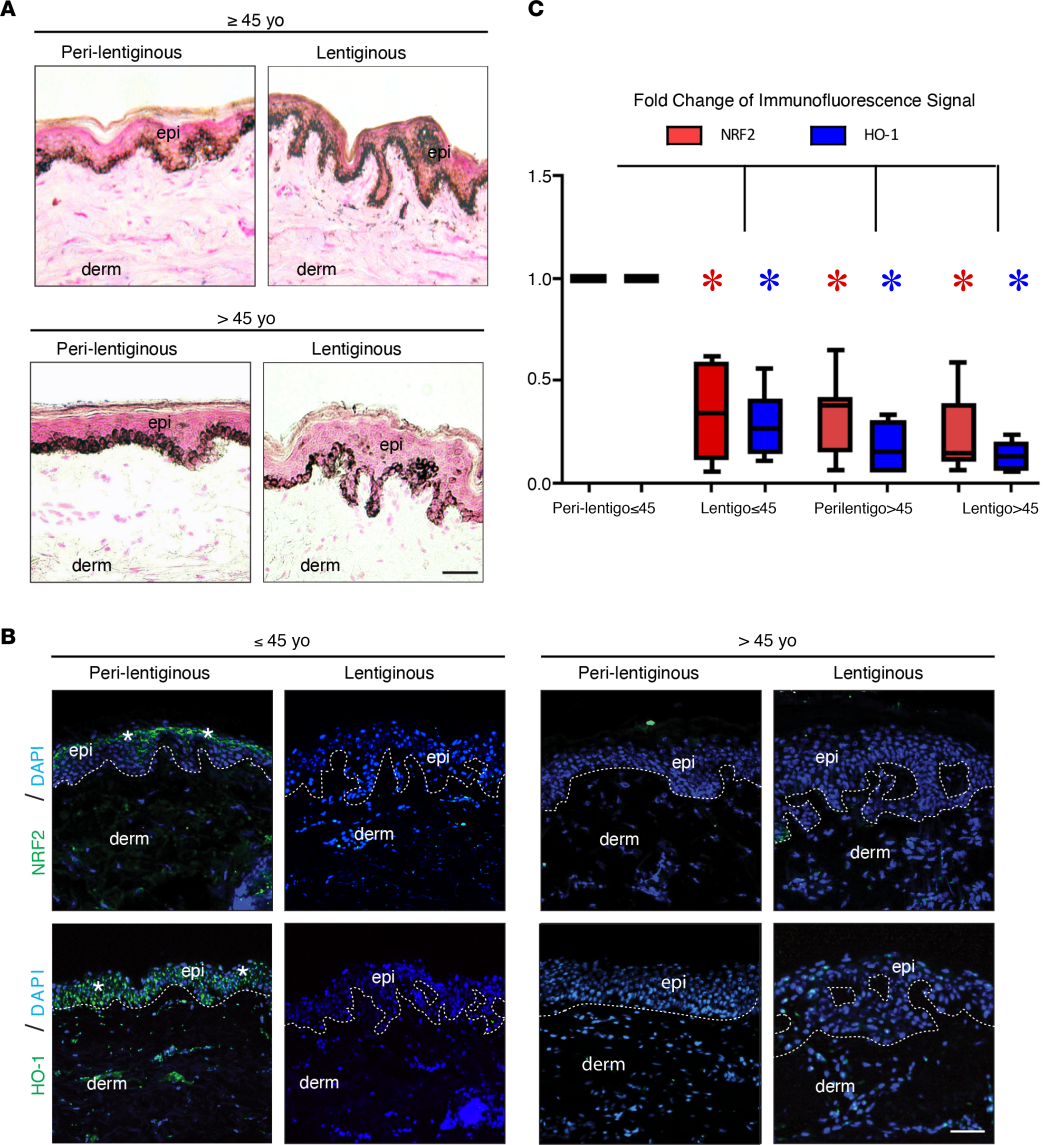
Solar lentigines and older photodamaged skin have lower levels of NRF2 and its target HO-1. (**A**) Representative Fontana-Masson (F&M) staining of perilentiginous and lentiginous skin from younger (≤ 45 years old [≤ 45 yo]) and older (> 45 yo) individuals. Scale bar: 50 μm. (**B**) Representative indirect immunofluorescence labeling of NRF2 and HO-1. DAPI, nuclear staining; epi, epidermis; derm, dermis. Dotted lines delineate the dermoepidermal junction. Scale bar: 50 μm. Asterisks mark areas of increased immunofluorescence signal. (**C**) Quantitation of immunofluorescence signal for perilentiginous younger (≤ 45) versus older (> 45) tissue labeled with antibodies against NRF2 or HO-1. Data represent mean ± SEM. *P < 0.05, between indicated groups as calculated by a 2-tailed unpaired Student’s *t* test. *P* values were corrected for multiple comparisons using a 2-stage linear step-up procedure of Benjamini, Krieger, and Yekutieli.

Chronic photodamage leads to the formation of hyperpigmented macules known as solar lentigines. However, the signaling pathways that contribute to the discrete areas of skin hyperpigmentation are not well defined. NRF2 signaling regulates transcriptional programs involved in adaption and survival of cells in the setting of oxidative stress, and oxidative stress occurs in the setting of photodamage. Thus, the expression levels of NRF2 and its target heme-oxygenase-1 (HO-1) were evaluated by immunofluorescence (IF) in skin biopsies from solar lentigines and surrounding (perilentiginous) skin of the dorsal forearms of 14 human subjects. Six subjects were ≤45 years of age (younger), and eight subjects were >45 years old (older) (Table 1). Using the classic histologic features of solar lentigines (e.g., club-shaped rete regions and pigmentation of the basal layer), the solar lentigines could be discriminated from surrounding perilentiginous skin (Figure 1A). The highest expression of NRF2 and HO-1 in the epidermis was in the perilentiginous skin from individuals ≤45 years old (Figure 1, B and C). In contrast, the expression of NRF2 and HO-1 was significantly reduced in the perilentiginous skin from individuals >45 years old, compared with the perilentiginous skin from individuals ≤45 years old (*P* < 0.05). In both younger and older individuals, there was also a significant decrease in expression of both NRF2 and HO-1 in lentiginous skin compared with nonlentiginous skin of the younger individuals (*P* < 0.05).

### NRF2 agonist treatment improves hyperpigmentation in photodamaged skin.

**Figure 2:**
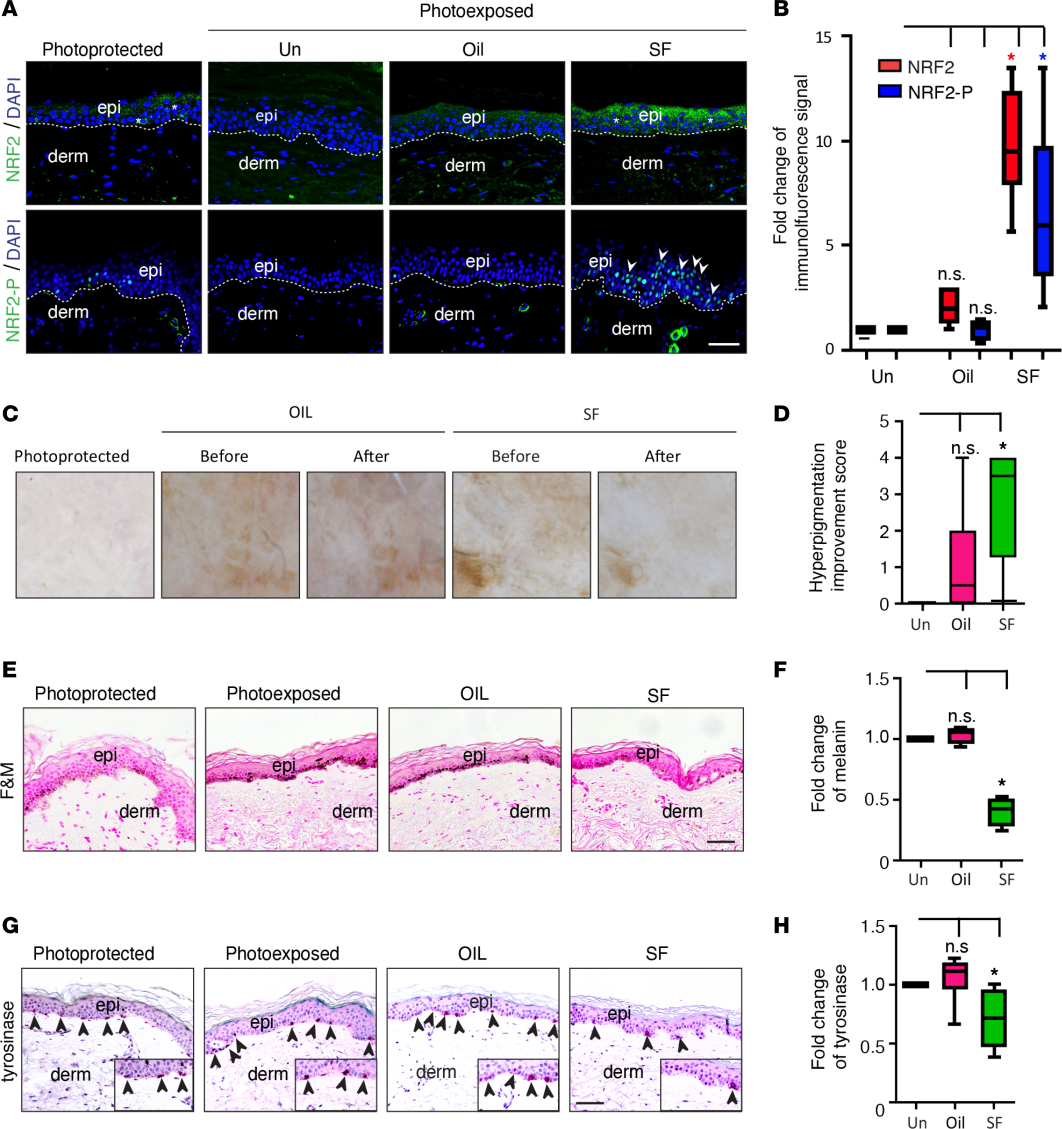
NRF2 agonist treatment corresponds to an improvement in hyperpigmentation in photodamaged human skin. Photoprotected (upper inner arm) and photoexposed skin (forearm) either received vehicle (OIL) or sulforaphane (SF) treatment. (**A**) Representative indirect immunofluorescence images of NRF2 and NRF2-P expression. DAPI, nuclear staining; epi, epidermis; derm, dermis; Un, untreated. Dotted lines delineate the dermoepidermal junction. Scale bar: 50 μm. Asterisks mark areas of increased immunofluorescence signal. Arrowheads denote nuclear NRF2-P labeling. (**B**) Immunofluorescence signal for NRF2 and NRF2-P (mean ± SEM). (**C**) Representative dermoscopy images (10-fold magnification). (**D**) Mean mottled hyperpigmentation improvement score (scale 0–4) ± SEM. *x^2^* (1, *n* = 6) = 4.3105, *P* = 0.037877. (**E**) Representative Fontana-Masson (F&M) staining. sc, stratum corneum, Scale bar: 50 μm. Black arrows indicate positive staining. (**F**) Quantitation of fold change in melanin per F&M staining (mean ± SEM). (**G**) Representative tyrosinase staining. Scale bar: 50 μm; 2-fold magnification (insets). Black arrows indicate positive staining. (**H**) Quantitation of fold change in tyrosinase staining (mean ± SEM). *P < 0.05, between indicated groups, as calculated by a (**B**) 2-tailed Student’s *t* test, (**D**) χ^2^ test, or (**F** and **H**) Mann Whitney *U* test. *P* values were corrected for multiple comparisons using either a 2-stage linear step-up procedure of Benjamini, Krieger, and Yekutieli (*t* test) or a Bonferroni correction (Mann Whitney *U* test).

Since there was reduced expression of NRF2 in photodamaged skin, particularly in lentiginous skin in older individuals (>45 years old), we next evaluated whether topical administration of the NRF2 agonist SF to photodamaged skin of older individuals had any therapeutic effect in normalizing NRF2 expression and reducing the mottled skin hyperpigmentation. Eight subjects (ages 52–77 years, mean age 60.1 years, Fitzpatrick skin phototypes I–III, Table 2) applied either broccoli sprout extract (BSE) containing 5 nM SF or vehicle (jojoba oil) alone in a blinded fashion to a 4 cm^2^ area of clinically similar photodamaged skin on either forearm as well as photoprotected skin on the upper inner arm daily for 7 days. The expression of total NRF2 and phosphorylated NRF2 (NRF2-P) by IF microscopy was detected at low baseline levels in photoprotected skin, suggesting some activity of the pathway, whereas the expression of total NRF2 and NRF2-P was undetectable in untreated photoexposed skin (Un) (Figure 2, A and B). There was significantly elevated IF expression and fold change of IF signal of NRF2 and especially NRF2-P in SF-treated skin compared with Un skin in 6 of the 8 individuals (data for these 6 responder individuals are shown in Figure 2). The mottled hyperpigmentation of the skin was also evaluated using a blinded hyperpigmentation improvement score. SF treatment of photoexposed skin resulted in an average improvement score of 2.8 ± 0.4 for mottled hyperpigmentation (Figure 2, C and D), an approximate 50% reduction of melanin deposition (Figure 2, E and F), and an approximate 30% reduction in fold change of tyrosinase expression (Figure 2, G and H), compared with Un or vehicle treatment (*P* < 0.05). In addition to the data for the 6 of 8 responders to the SF treatment (Figure 2), there was no evidence of increased total NRF2 or NRF2-P expression in SF-treated photoexposed skin in 2 of the 8 individuals (Supplemental Figure 1A; supplemental material available online with this article; https://doi.org/10.1172/jci.insight.139342DS1). In these 2 nonresponder subjects, there was also no significant improvement in mottled hyperpigmentation (Supplemental Figure 1B) or difference in melanin deposition (Supplemental Figure 1C) following SF treatment compared with vehicle or Un controls (Supplemental Figure 1, B and C). These results further indicate that the effects of SF in reducing skin hyperpigmentation and melanin deposition in the responder individuals were likely dependent upon SF-induced NRF2 and NRF2-P expression.

### NRF2 agonist treatment abrogates UVB-induced hyperpigmentation in mice.

**Figure 3:**
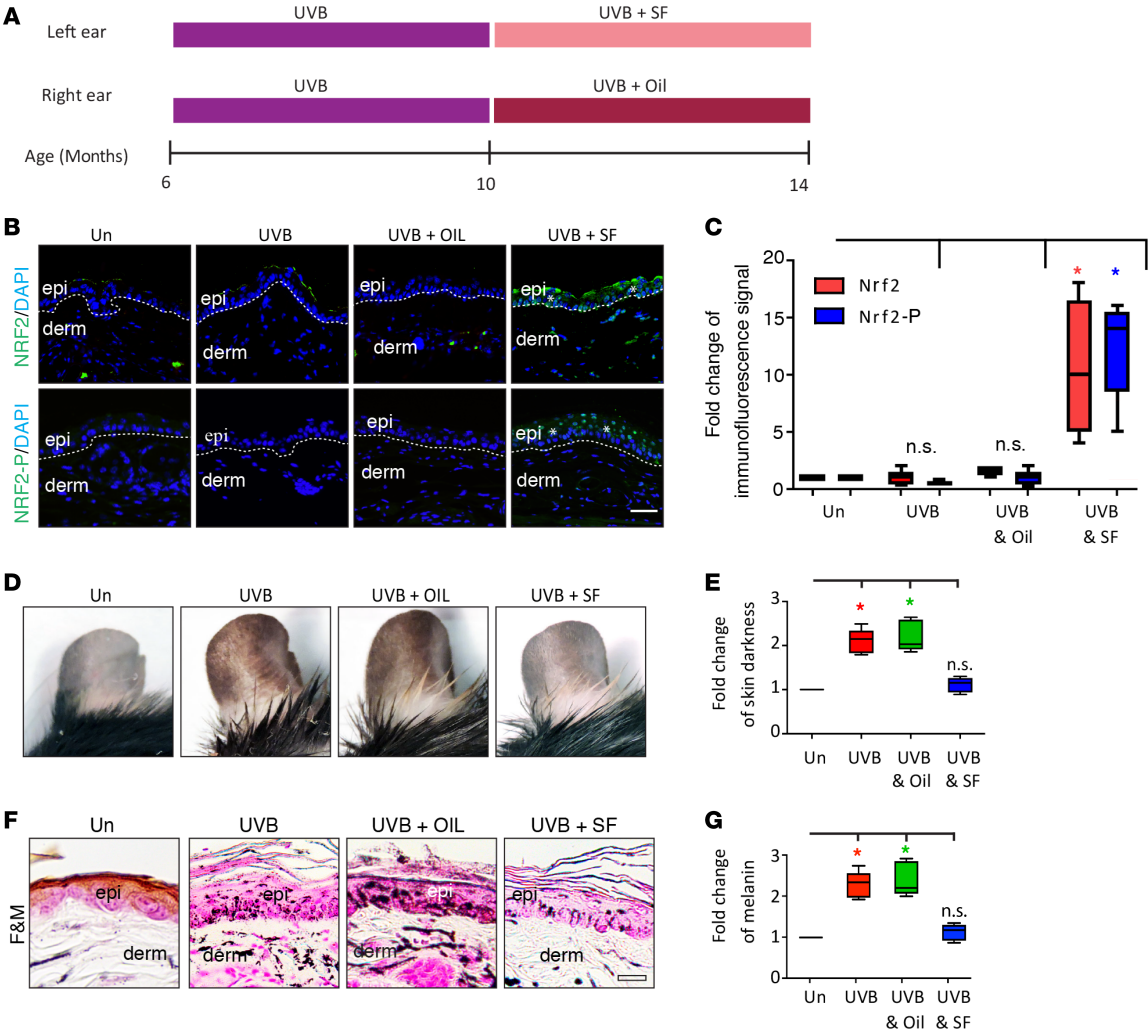
NRF2 agonist treatment reduces UVB-induced ear skin pigmentation in mice. (**A**) Schematic of treatment regimen for WT mice that were either untreated (Un) or received UVB exposure alone (UVB), UVB + vehicle treatment (UVB+OIL), or UVB + NRF2 agonist (SF) treatment (UVB+SF). (**B**) Representative indirect immunofluorescence. DAPI, nuclear staining; epi, epidermis; derm, dermis. Dotted lines delineate the dermoepidermal junction. Asterisks mark areas of increased immunofluorescence signal. Scale bar: 50 μm. (**C**) Fold change of immunofluorescence signal for NRF2 and NRF2-P (mean ± SEM). (**D**) Representative images of ear skin. (**E**) Fold change of skin darkness (mean ± SEM). (**F**) Representative Fontana-Masson (F&M) staining. Scale bar: 50 μm. (**G**) Fold change of melanin ± SEM. *P < 0.05, between indicated groups as calculated by a (**C**) 2-tailed unpaired Student’s *t* test or (**E** and **G**) a Mann Whitney *U* test. *P* values for were corrected for multiple comparisons using either a 2-stage linear step-up procedure of Benjamini, Krieger, and Yekutieli (*t* test) or a Bonferroni correction (Mann Whitney *U* test).

To corroborate these findings in human subjects shown in Figure 2 and Supplemental Figure 1, a mouse model of UVB-induced hyperpigmentation was used. To first establish skin hyperpigmentation, mouse ears of older (6 month old) male C57BL/6 WT mice were exposed to 80 mJ/cm^2^ UVB daily 5 times a week for 4 total weeks. UVB exposure was then continued for an additional 4 weeks, with topical application of SF (1 μM) (UVB+SF) to one ear prior to UVB treatment or vehicle (UVB+Oil) to the other ear of each mouse so that every mouse served as its own control (Figure 3A). UVB treatment was continued during the topical treatment period to reflect the continued everyday UV exposure that our subjects experienced during the trial. An age-matched control group that received no UVB exposure and no treatment (Un group) was also included as well as an age-matched control group that received UVB exposure without any treatment (UVB group). NRF2 and NRF2-P expression in the skin by IF microscopy was assessed as in Figure 2A and B (Figure 3, B and C). Un, UVB, and UVB+Oil treatment resulted in no significant increase in expression of NRF2 or NRF2-P. In contrast, UVB+SF treatment increased NRF2 (~5-fold, *P* < 0.05) and NRF2-P (~10-fold, *P* < 0.05) significantly. Despite the lack of increased NRF2 and NRF2-P, UVB and UVB+Oil treatment resulted in increased skin pigmentation (2.2 ± 0.2–fold and 2.5 ± 0.3–fold increase of skin pigmentation, respectively, *P* < 0.05 for both) and exhibited a marked increase of melanin deposition (Figure 3, D and E). In contrast, in the UVB+SF group, the increased skin pigmentation and melanin deposition were completely abrogated, as there was no significant change in skin pigmentation or melanin deposition compared with the Un group (Figure 3, D–G). Accordingly, in the UVB+SF group, there was more than a 50% decrease in fold change of skin darkness and in melanin deposition compared with the UVB+Oil group (*P* < 0.0001, for both).

### NRF2 agonist treatment prevents UVB-induced hyperpigmentation in mice.

**Figure 4:**
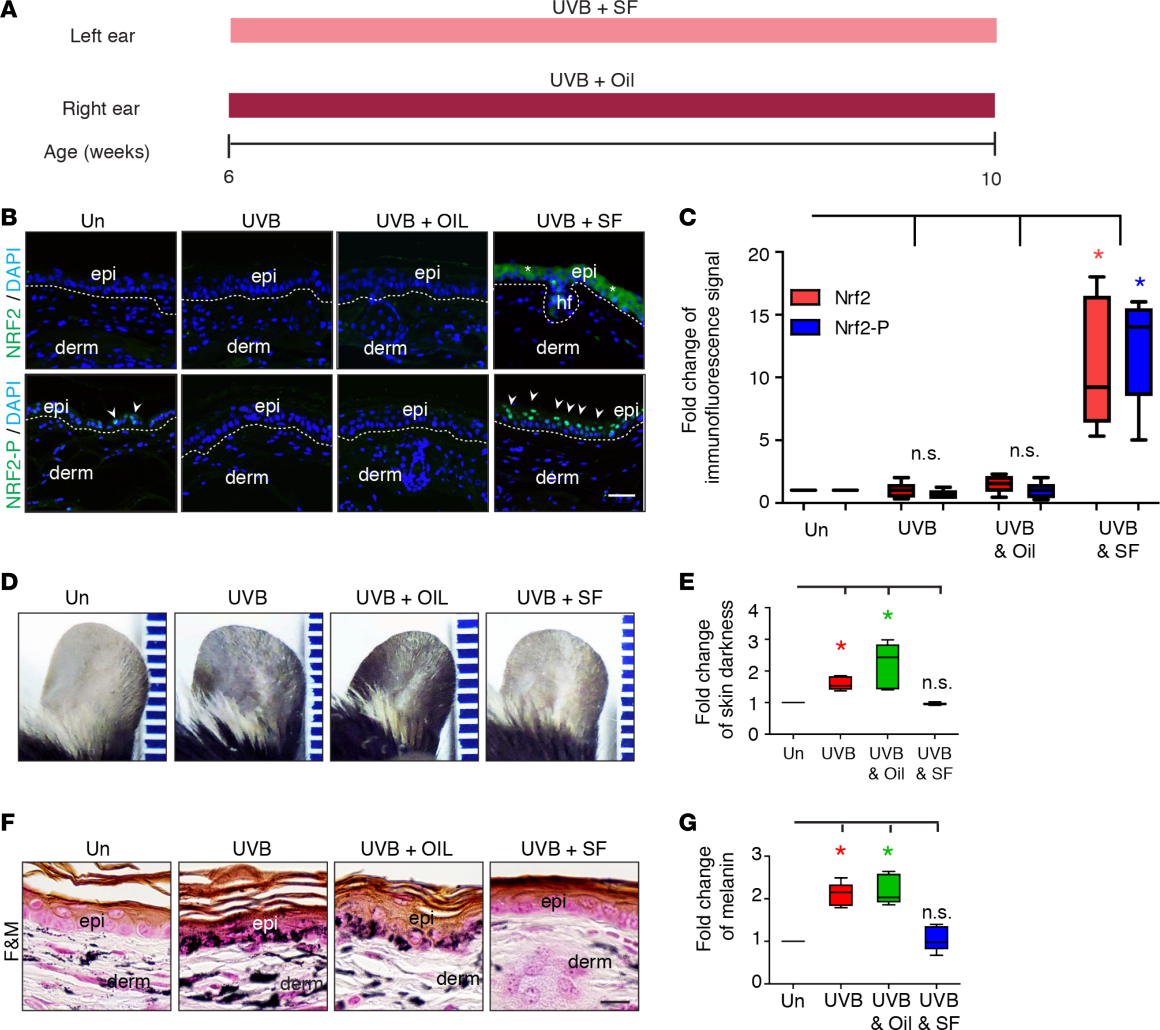
NRF2 agonist treatment prevents UVB-induced skin ear pigmentation in mice. (**A**) Schematic of preventative treatment regimen for WT mice that were either untreated (Un) or received UVB exposure alone (UVB), UVB + vehicle treatment (UVB+OIL), or UVB + NRF2 agonist (SF) treatment (UVB+SF). (**B**) Representative indirect immunofluorescence. Scale bar: 50 μm. DAPI, nuclear staining; epi, epidermis; derm, dermis; hf, hair follicle. Dotted lines mark the dermoepidermal junction. Arrowheads mark areas of increased immunofluorescence signal. (**C**) Fold change of immunofluorescence signal for NRF2 and NRF2-P (mean ± SEM). (**D**) Representative images of ear skin. (**E**) Fold change of skin darkness (mean ± SEM). (**F**) Representative Fontana-Masson (F&M) staining. Scale bar: 50 μm. (**G**) Fold change of melanin (mean ± SEM). *P < 0.05, between indicated groups as calculated by (**C**) 2-tailed unpaired Student’s *t* test or (**E** and **G**) a Mann Whitney *U* test. *P* values for were corrected for multiple comparisons using either a 2-stage linear step-up procedure of Benjamini, Krieger, and Yekutieli (*t* test) or a Bonferroni correction (Mann Whitney *U* test).

Next, the ability of SF to prevent UVB-induced hyperpigmentation was evaluated by using a similar model as that shown in Figure 3 but without the first 4 weeks of UVB exposure. In this experiment, younger (6 weeks old) C57BL/6 WT mice were exposed to UVB daily 5 times a week for 4 weeks, with topical application of SF (1 μM) (UVB+SF) prior to UVB treatment on one ear or vehicle (UVB+Oil) on the other ear of each mouse, so that every mouse served as its own control (Figure 4A). Un, UVB, and UVB+Oil treatment resulted in no induction of expression of NRF2 or NRF2-P above baseline, as determined by IF microscopy (Figure 4, B and C). In contrast, UVB+SF had significantly marked increased expression (~10-fold) of both NRF2 and NRF2-P (*P* < 0.05). Similar to our previous results in Figure 3, UVB and UVB+Oil treatment significantly increased skin pigmentation (1.6 ± 0.1–fold and 2.2 ± 0.3–fold increase, respectively) (Figure 4, D and E) and melanin deposition (Figure 4, F and G) (*P* < 0.05, for both), whereas UVB+SF treatment resulted in no significant change in skin darkness or melanin deposition compared with Un skin. In addition, UVB+SF treatment resulted in more than a 50% decrease in skin pigmentation and melanin deposition compared with UVB+Oil treatment (*P* < 0.0001), indicating that SF could prevent UVB-induced skin pigmentation.

### NRF2 agonist treatment is specific to its effect on the NRF2 pathway.

To determine whether the preventative effect of SF on UV-induced hyperpigmentation was due to the effect of SF on activating the NRF2 pathway or an alternative mechanism, the experiment in Figure 4 was performed in *Nrf2^–/–^* mice. Similar to that in WT mice in Figure 4, UVB and UVB+Oil treatment resulted in significantly increased skin pigmentation compared with Un mice (Supplemental Figure 2, A and B). However, in contrast to WT mice, UVB+SF treatment of *Nrf2^–/–^* mice resulted in significantly increased skin pigmentation compared with Un *Nrf2^–/–^* mice that was not significantly different than the skin pigmentation in UVB or UVB+Oil treated *Nrf2^–/–^* mice. Therefore, the effect of SF on decreasing skin pigmentation did not correlate with decreased tyrosinase activity in *Nrf2^–/–^* mice, which indicates that the effect of SF was due to its specific activity on the NRF2 pathway.

### Keratinocyte expression of IL-6Rα is required for NRF2 agonist activity and NRF2 expression.

**Figure 5:**
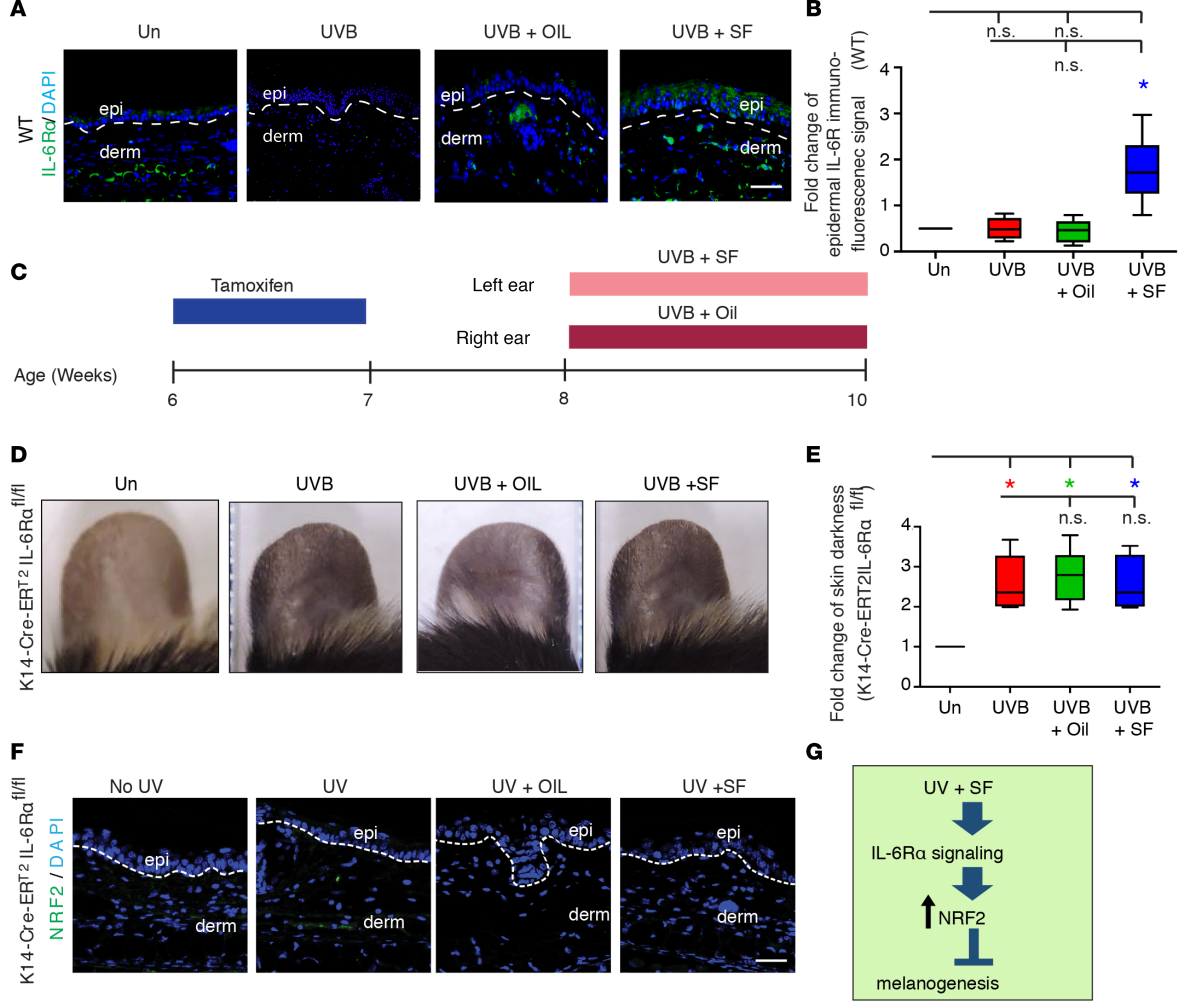
NRF2 agonist prevention of UVB-induced skin ear pigmentation in mice is specific to NRF2 signaling and requires keratinocyte-intrinsic IL-6Rα signaling. (**A**) Representative indirect immunofluorescence for IL-6Rα. DAPI, nuclear staining; epi, epidermis; derm, dermis. Dotted lines delineate the dermoepidermal junction. Scale bar: 50 μm. (**B**) Fold change of immunofluorescence signal for IL-6Rα (mean ± SEM). (**C**) Schematic of preventative treatment regimen for K14-Cre-ER^T2^IL-6Rα^fl/fl^ mice that were either untreated (Un) or received UVB exposure alone (UVB), UVB + vehicle treatment (UVB+OIL), or UVB + NRF2 agonist (SF) treatment (UVB+SF). (**D**) Representative images of ear skin. (**E**) Fold change of skin darkness (mean ± SEM). (**F**) Representative indirect immunofluorescence for NRF2. Scale bar: 50 μm. (**G**) Proposed mechanism. *P < 0.05, between indicated groups as calculated by a Mann-Whitney *U* test. *P* values were corrected for multiple comparisons using a Bonferroni correction.

Skin pigmentation involves complex interactions between keratinocytes and melanocytes. Prior in vitro experiments demonstrated that an extract of the plant *Lepidium apetalum* (ELA), belonging to the *Brassicaeae* family, decreased UV-induced melanogenesis secondary to IL-6 production by keratinocytes (27). Notably, other plants belonging to the *Brassicaceae* family contain high levels of NRF2 inducers (28). Although a role for NRF2 signaling was not evaluated in this prior report, it could be that IL-6 was involved in SF-induced NRF2-dependent decreased hyperpigmentation following UVB exposure. Given that NRF2 and NRF2-P expression was observed in the epidermal keratinocytes after SF exposure (Figure 3, B and C, and Figure 4, B and C), we hypothesized that if IL-6 contributed to NRF2 signaling or expression (and subsequent decreased pigmentation), it would be due to a direct effect of IL-6 on the keratinocytes. Therefore, the epidermal expression of the IL-6 receptor α (IL-6Rα) in keratinocytes was first evaluated by IF microscopy in the same experimental groups as in Supplemental Figure 2, A and B (Figure 5, A and B). UV and UV+Oil treatment did not upregulate the epidermal expression of IL-6Rα compared with Un. However, UV+SF treatment resulted in more than 3-fold upregulation of epidermal IL-6Rα expression compared with Un, indicating that SF treatment induces expression of IL-6Rα by keratinocytes, which would increase their sensitivity to IL-6.

Next, to determine whether the SF-induced IL-6Rα–intrinsic expression specifically by keratinocytes contributed to decreased skin pigmentation, we generated a mouse line with tamoxifen-inducible IL-6Rα deletion in keratinocytes (i.e., K14-Cre-ER^T2^IL-6Rα^fl/fl^ mice). K14-Cre-ER^T2^IL-6Rα^fl/fl^ mice were first treated with tamoxifen to delete IL-6Rα in keratinocytes, and then the UVB-induced skin pigmentation model was performed as in Figure 4 and Supplemental Figure 1 (Figure 5C). In the K14-Cre-ER^T2^IL-6Rα^fl/fl^ mice, the UVB exposure led to 2-fold increased skin pigmentation in only 2 weeks (compared with 4 weeks for C57BL/6 WT mice), and the experiment was thus only conducted for 2 weeks. Of note, we have found that a shorter course of SF is sufficient to robustly induce NRF2 (7 days in human skin and 4 days in mouse skin) (29, 30). UVB, UVB+Oil, and UVB+SF treatment all resulted in the development of increased skin pigmentation (Figure 5, D and E). The inability of SF to reduce the skin pigmentation in K14-Cre-ER^T2^IL-6Rα^fl/fl^ mice was similar to the findings in Nrf2^–/–^ mice (Supplemental Figure 2, A and B). Thus, the expression of NRF2 was evaluated in the ear skin in all experimental groups. Remarkably, Un and treatment with UVB, UVB+Oil, and UVB+SF did not induce NRF2 expression in K14-Cre-ER^T2^IL-6Rα^fl/fl^ mice (Figure 5F). Taken together, the therapeutic effect of SF on reducing UVB-induced skin pigmentation was therefore dependent on keratinocyte-intrinsic IL-6Rα signaling that upregulated NRF2, which led to inhibition of melanogenesis (Figure 5G).

## Discussion

Chronic UVR exposure leads to mottled hyperpigmentation in photoaged skin, including the formation of solar lentigines. Given that the mechanisms that contribute to the development of solar lentigines are not well understood, we evaluated the role and therapeutic potential of NRF2 signaling because of its known activity in conditions associated with oxidative stress. Analysis of NRF2 signaling in photodamaged human skin revealed that NRF2 signaling was dysregulated in solar lentigines. SF is known to have several non-NRF2–mediated targets, such as NF-κB and AP-1 (31). However, our findings in both human and mouse studies reported here suggest that the negative regulation of UV-mediated hyperpigmentation observed following SF treatment is occurring in an NRF2-dependent fashion. Topical application of the NRF2 inducer SF to photodamaged human skin triggered NRF2 signaling and lightened mottled hyperpigmentation in 8 of our 6 subjects. The 2 subjects that did not activate NRF2 following topical application of SF also did not show an improvement of mottled pigmentation. These findings were corroborated in a mouse model of UVB-induced hyperpigmentation, whereby topical SF treatment prevented and treated UVB-induced hyperpigmentation of mouse ear skin. By contrast, comparable topical SF treatments of UVB-induced hyperpigmentation of the ear skin of *Nrf2^–/–^* mice had no inhibition of the hyperpigmentation, indicating that the effect of SF on UVB-induced hyperpigmentation occurred specifically through its activity on the NRF2 pathway. Finally, in mouse skin in which the IL-6Rα was conditionally and specifically deleted in keratinocytes (tamoxifen exposure of K14-Cre-ER^T2^IL-6Rα^fl/fl^ mice), topical SF treatment failed to activate NRF2 signaling or inhibit pigmentation following UVB exposure. Collectively, our findings provide an explanation for several previously reported results involving the mechanisms associated with skin hyperpigmentation and provide the potential for therapeutic intervention of aberrant skin hyperpigmentation in humans.

First, under homeostatic conditions, NRF2 is constitutively expressed in all skin cell types, and its activity is regulated by protein turnover via KEAP1-mediated ubiquitination and proteosomal degradation. Reactive cysteines in KEAP1 effectively serve as cellular redox sensors, and their modification by ROS and electrophiles lead to a conformational change, allowing the release, stabilization, and nuclear translocation of NRF2 (17). The autophagy receptor p62 can also interact with the NRF2-binding site on KEAP1, competing with the interaction between NRF2 and KEAP1. This results in stabilization of NRF2 and transcriptional activation of NRF2 target genes, which are instrumental for antioxidant, antiinflammatory, and detoxification of cellular defense responses (18). Thus, the NRF2/KEAP1 redox sensitive signaling axis is primed to rapidly react to electrophilic and oxidative cellular stresses, including chronic UVA exposure and subphysiological doses of UVB (19, 20). Our results indicate that NRF2 signaling is involved UV-induced hyperpigmentation in both human and mouse skin, providing direct in vivo evidence of how NRF2 is involved in response to oxidative stress associated with photodamage and chronic UV exposure.

Second, progressive derangement of NRF2 signaling has been hypothesized to be a critical factor driving the age-related decline in antioxidant defense of several organ systems (11, 21). With respect to the skin, the photoprotective effects of pharmacological activation of NRF2 have been demonstrated in cultured human cells, reconstructed human skin, and mouse skin (22–24). Alterations in NRF2 signaling have been observed in stress-induced loss of pigmentation disorders of both the skin and hair follicles, such as vitiligo and hair graying, respectively (25). Notably, the complex crosstalk between keratinocytes and melanocytes that mediates UVB-induced pigmentation has been shown to be affected by NRF2 signaling via an ROS-dependent mechanism in cultured cells (26). Studies by Shin et al., which were conducted in cultured normal melanocytes transduced with a recombinant adenovirus expressing NRF2, have demonstrated that NRF2 negatively regulates melanogenesis by modulating PI3K/Akt signaling (32). Similar to our findings in mouse skin, the effect of NRF2 on tyrosinase levels of expression and activity occurred relatively quickly, i.e., within 3 days. Our results build on these in vitro findings and provide in vivo evidence of how NRF2 signaling is involved in hyperpigmentation of the skin and how targeting NRF2 signaling with SF could serve as a potential therapeutic agent for skin hyperpigmentation associated with photodamage.

Third, there is growing evidence that crosstalk between TLR signaling and NRF2 signaling serves as a bridge between the oxidative stress response and immune regulation through modulation of inflammation (33). TLR signaling activates NRF2 signaling by reducing KEAP1 levels via the induction of p62 (34). Thus, the NRF2/KEAP1 redox-sensitive signaling axis is primed to rapidly react to electrophilic and oxidative cellular stresses, including chronic UV exposure, including UVA and subphysiological doses of UVB (19, 20), and also serve as a negative feedback mechanism for TLR-mediated inflammation. Interestingly, TLR-mediated induction of IL-6 production has been linked to prevention of UV-induced hyperpigmentation. Following UVB exposure, IL-6 is rapidly induced in the skin primarily through TLR signaling (35). The acceleration of hyperpigmentation observed in UV-treated K14-Cre-ER^T2^IL-6Rα^fl/fl^ mice, compared with WT mice (2 weeks versus 4 weeks), further supports a critical role of IL-6R signaling in the UV response, including UV-induced hyperpigmentation. We found that topical SF failed to inhibit UVB-induced ear skin hyperpigmentation of K14-Cre-ER^T2^IL-6Rα^fl/fl^ mice (Figure 5, D and E), and this provides a mechanistic explanation of how IL-6*^–/–^* mice are more sensitive to UVB irradiation (36). Moreover, there was a lack of upregulation of NRF2 in the skin of K14-Cre-ER^T2^IL-6Rα^fl/fl^ mice (Figure 5F), suggesting that IL-6 was a key inducer of NRF2.

Finally, a prior in vitro study found that an extract of the plant *Lepidium apetalum* belonging to the *Brassicaeae* family reduced UV-induced melanogenesis by first inducing IL-6 production by keratinocytes, which subsequently downregulated the transcription factor microphthalmia-associated transcription factor (MITF) in melanocytes, and ultimately leading to tyrosinase gene expression and melanocyte differentiation (27). Although isothiocyanates derived from cruciferous plants belonging to the *Brassicaceae* family are potent inducers of NRF2 (28), a role for NRF2 signaling was not evaluated. Our results build on these in vitro studies and demonstrate in vivo that topical administration of SF, another potent inducer of NRF2 signaling, was able to improve photodamage-associated hyperpigmentation in human skin and UV-induced hyperpigmentation in mouse skin in a mechanism involving NRF2 signaling and IL-6/IL-6Rα signaling.

There are some limitations. First, a more complete assessment of the potential effect of NRF2 on other aspects of the complex regulation of skin pigmentation following UV exposure, including the regulation of melanosome transport and degradation, would further delineate the function of NRF2 signaling on skin pigmentation. Moreover, the role of NRF2 as a well-known promoter of keratinocyte differentiation and skin turnover may help explain our findings of decreased melanin and mottled hyperpigmentation of human skin after only 7 days of SF treatment (37). However, these studies are beyond the scope of this initial report. Second, further clinical studies with an increased number of human subjects, longer treatment regimens, and additional body sites are needed to verify the generalizability of our findings and to further assess the long-term effects of NRF2 activation on photoaging. Third, a clinical study examining the effects of pretreatment with SF prior to UVB exposure in a younger patient cohort might also be warranted to evaluate whether SF treatment can protect against UVB-induced hyperpigmentation in human skin. Finally, it is tempting to speculate the NRF2 signaling might be involved in other skin hyperpigmentation disorders that are induced or exacerbated by UV exposure as well as in skin carcinogenesis. These limitations will be addressed in our future research work.

Taken together, the NRF2 signaling pathway was identified as an important signaling pathway involved in the pathogenesis of hyperpigmentation associated with photodamage in human skin and in UV-induced hyperpigmentation in mouse skin in vivo. Our findings further provide key insights into how NRF2 signaling is linked with IL-6/IL-6Rα signaling in UV-induced skin pigmentation. Most importantly, treatment of human or mouse skin hyperpigmentation with SF provided the proof of concept for targeting the NRF2 pathway as a potentially novel therapeutic intervention against hyperpigmentation in response to photoaging or UV exposure.

## Methods

### Human subjects and SF treatment of human skin.

To determine the expression of NRF2 and HO-1, shave biopsies of lentiginous skin with surrounding clinically normal appearing skin (perilentiginous skin) were obtained from 14 individuals (Table 1). For the topical SF treatment, 7 subjects (Table 2) applied BSE containing SF (5 nM, LKT Laboratories Inc.) daily or vehicle oil (jojoba oil; MP Biomedical LLC) in a blinded fashion to 4 cm^2^ clinically similar areas of photodamaged skin on either forearm for 7 days. The left arm was chosen for treatment with the BSE, as there is typically more photodamage on the left arm due to chronic sun exposure through the car window while driving in the US. A photoprotected area of skin on an upper inner arm was also treated. Following 7 days of treatments, 4 mm punch biopsies from individuals were obtained for further analyses. During the visits before and after treatment, dermoscopy images were taken with polarized and nonpolarized light following application of a drop of mineral oil using a DermLite Foto II Pro dermoscopy lens attached to a DSLR Nikon D300s camera. Study subjects were instructed to avoid food and supplements containing SF and to refrain from applying any topicals to marked treatment areas for the duration of the study.

### Mice.

WT and *Nrf2^–/–^* (Nfe2l2^tm1Ywk^) male C57BL/6 mice were obtained from The Jackson Laboratory. K14-Cre-ER^T2^IL-6Rα^fl/fl^ mice were generated by breeding K14-Cre-ER^T2^ (Tg[KRT14-cre/ERT]20Efu/J) mice with IL-6Rα^fl/fl^ (B6;SJL-Il6ra^tm1.1Drew^/J) mice (both obtained from The Jackson Laboratory). K14-Cre-ER^T2^ mice have tamoxifen-inducible expression of Cre-recombinase (Cre) specifically in keratinocytes. IL-6Rα^fl/fl^ mice have Cre-binding *loxP* sequences that flank exons 4–6 of the *Il6ra* gene. K14-Cre-ER^T2^ × IL-6Rα^fl/fl^ mice were treated systemically with 100 μL of 10 mg/mL tamoxifen. Confirmation of deletion of IL-6Rα was performed via qPCR of mouse tail genomic DNA from K14-Cre-ER^T2^ × IL-6Rα^fl/fl^ mice with or without tamoxifen treatment, whereby primers were designed to detect the presence of Cre, floxed IL-6Rα, WT IL-6Rα, and deletion of floxed IL-6Rα (Transnetyx).

### Mouse model of UVB-induced hyperpigmentation and SF treatment.

For topical SF treatment, 6-month-old WT and *Nrf2^–/–^* C57BL/6 male mice were exposed to 80 mJ/cm^2^ UVB (range 290–315 nm) using a Philips TL Broadband UVB lamp (TL 20W/12 RS SLV/25, Philips) in ventilated cabinets equipped with UVB lamps once daily 5 times a week for 4 weeks. For the next 4 weeks of UVB treatment, just prior to the UVB treatment, mice were anesthetized (2% isoflurane), and SF (1 μM; LKT Laboratories Inc.) was topically applied to the right ear (UVB+SF), while the left ear received either vehicle oil (jojoba oil, MP Biomedical LLC) (UVB+Oil) or no topical treatment (UVB). For the topical SF preventative treatment, 6-week-old WT, *Nrf2^–/–^*, or K14-Cre-ER^T2^IL-6Rα^fl/fl^ (previously treated with tamoxifen [1 mM] i.p. daily for 5 days) C57BL/6 male mice were exposed to 80 mJ/cm^2^ UVB daily 5 times a week for 4 weeks and, in the anesthetized mice just prior to UVB exposure, topical SF was applied to the right ear (UVB+SF), while the left ear received either vehicle oil (jojoba oil, MP Biomedical LLC) (UVB+Oil) or no topical treatment (UVB). In all experiments, an additional Un control group of age-matched male mice that received no UVB treatment and no topical treatment was included.

### Histologic analysis, IF, and immunohistochemistry.

Human skin biopsy specimens and mouse ear specimens were fixed in formalin (10%) and embedded in paraffin. 8 μm sections were cut on a microtome in the same orientation. Skin architecture, melanin deposition, and melanocytes were assessed following H&E and Fontana-Masson (F&M) staining, respectively. Tissue was also incubated with primary antibodies and Alexa Fluor–conjugated secondary antibodies for indirect IF. The primary antibodies used in this study included rabbit polyclonal antibodies against NRF2 (sc-13032; Santa Cruz Biotechnology), P-NRF2 (bs-2013R; Bioss Antibodies), and HO-1 (NB110-57028; Novus Biologicals). Immunohistochemistry for tyrosinase expression was performed by the Johns Hopkins Reference Histology and Immunopathology Core, according to guidelines for clinical specimens. Bright-field and IF microscopy images were obtained using a Leica DFC495 microscope (Leica).

### Skin pigmentation assessment.

Digital images were obtained from human and mouse ear skin. For both human and mouse skin, baseline and posttreatment images were assessed using blinded clinical scoring of mottled hyperpigmentation (scale 0–4; 0, no improvement; 1, minimal improvement; 3, moderate improvement; 4, clear or 100% improvement.)

### Statistics.

A 2-tailed Student’s *t* test or Mann-Whitney *U* test was used for parametric and nonparametric numerical data comparisons, respectively, as indicated in figure legends. Corrections for the false discovery rate were applied to account for multiple comparisons as follows. For Mann Whitney *U* tests, a Bonferroni correction was applied. For 2-tailed Student’s *t* test, a 2-stage linear step-up procedure of Benjamini, Krieger, and Yekutieli was performed. A χ^2^ test was used for nonparametric, noncontinuous data as indicated in figure legends. All data analysis was performed using GraphPad Prism version 8. A *P* value of less than 0.05 was considered significant. The quantification of IF signals, F&M staining, and images of skin darkness were performed in a blinded fashion using the image analysis software program ImageJ (NIH).

### Study approval.

All research in human subjects was approved by the Johns Hopkins Institutional Review Board. All patients were enrolled using an approved informed consent form. All mice were bred and maintained under specific pathogen–free conditions at an American Association for the Accreditation of Laboratory Animal Care–accredited animal facility at Johns Hopkins University and were housed according to procedures described in the *Guide for the Care and Use of Laboratory Animals* (National Academies Press, 2011). All animal procedures were approved by the Johns Hopkins University Animal Use and Care Committee.

## Author contributions

MLK, RJM, ASB, MM, LL, CAD, and BLP performed experiments and analyzed data. MLK, RW, and ALC conducted all studies with the human subjects. MLK, RJM, CAD, ASB, NKA, LSM, ALC, and SK conceived the study, interpreted data, and wrote the manuscript.

## Supplementary Material

supplemental data

## Figures and Tables

**Table 1:**
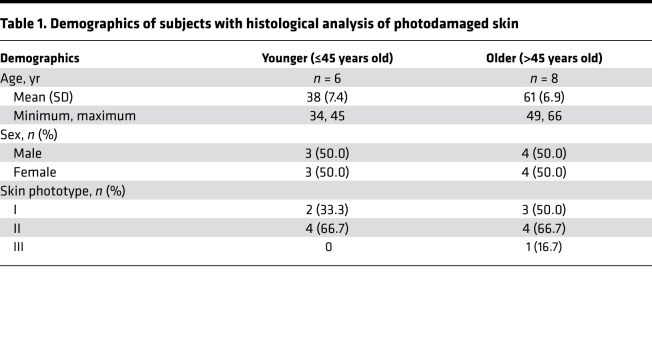
Demographics of subjects with histological analysis of photodamaged skin

**Table 2:**
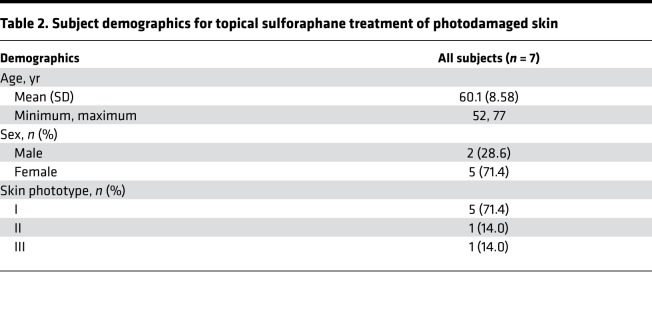
Subject demographics for topical sulforaphane treatment of photodamaged skin

## Version 1 10/01/2020

In-Press Preview

## Version 2 10/15/2020

Electronic publication
