# Supplementary material for: Pathogenic and therapeutic role for NRF2 signaling in ultraviolet light–induced skin pigmentation
Source: JCI Insight. 2020 Oct 15;5(20):e139342. doi: 10.1172/jci.insight.139342 (PMC7605539; doi:10.1172/jci.insight.139342)
Supplement: supplemental data [file jciinsight-5-139342-s247.pdf]

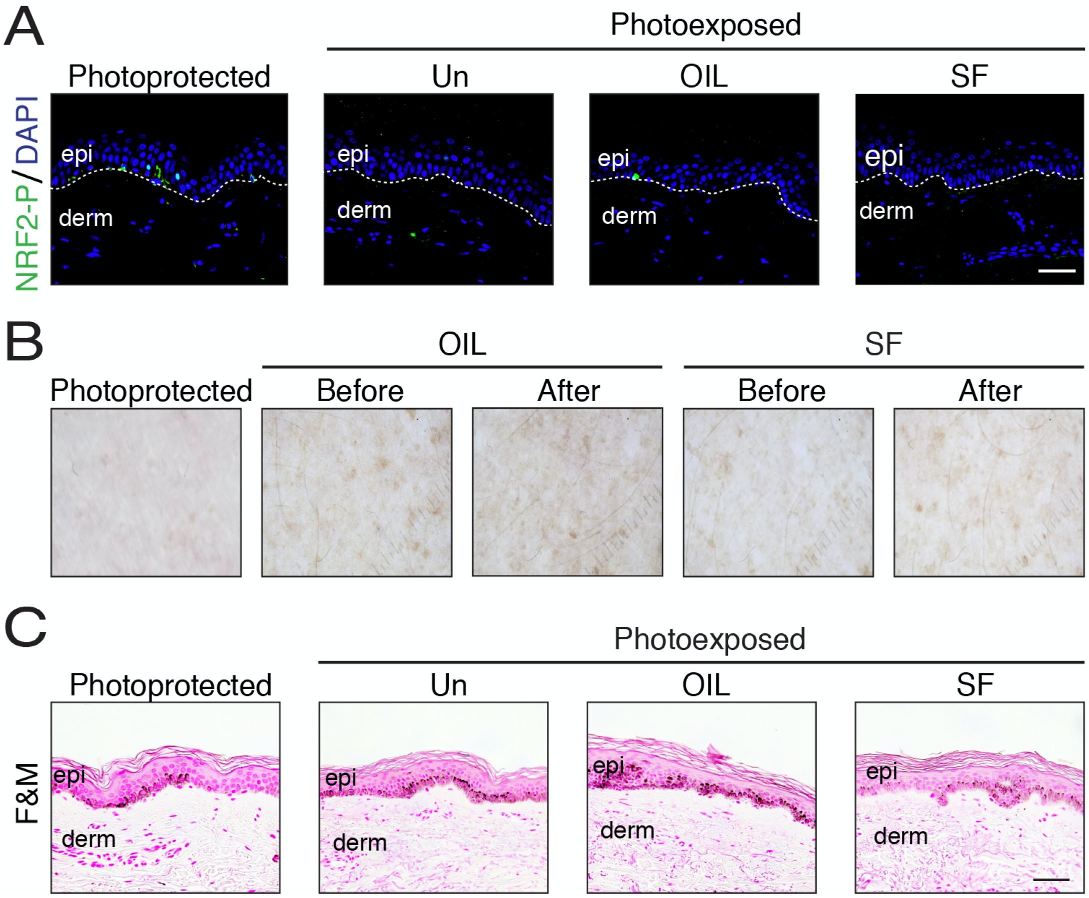

Figure S1. Lack of NRF2 induction in response to NRF2 agonist treatment corresponds to no reduction in hyperpigmentation in photodamaged skin. Photoprotected and photoexposed skin either received vehicle (OIL) or sulforaphane (SF) treatment. (**A**) Representative indirect immunofluorescence for NRF2 and NRF2-P. DAPI, nuclear staining; epi, epidermis; derm, dermis. Dotted lines delineate the dermoepidermal junction. Scale bar = 50 µm. Asterisks mark areas of increased immunofluorescence signal. (**B**) Representative dermoscopy images. (**C**) Representative Fontana-Mason (F&M) staining. sc, stratum corneum; epi, epidermis; derm, dermis. Scale bar = 50 µm.

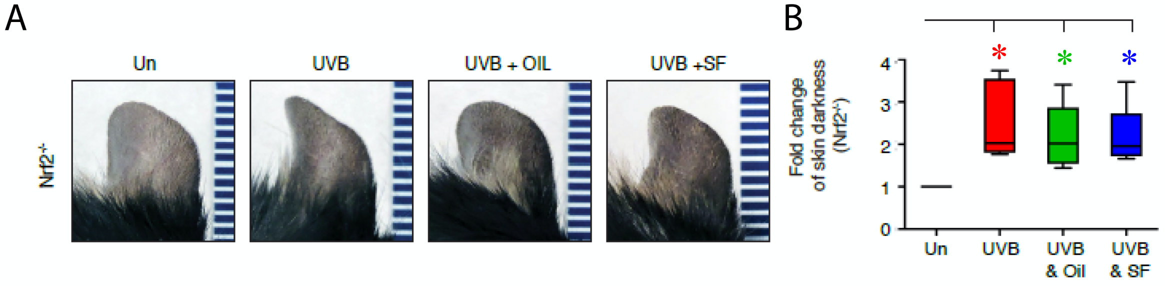

Figure S2. NRF2 agonist prevention of UVB-induced skin ear pigmentation in mice is specific to NRF2 signaling. (**A**) Schematic of preventative treatment regimen for NRF2$^{-/-}$ mice that were either Unreated (*Un*) or received UVB exposure alone (*UVB*), UVB + vehicle treatment (*UVB+OIL*) UVB + NRF2 agonist (SF) treatment (*UVB+SF*). (**B**) Mean fold change of skin darkness ± s.e.m. Mean fold change ± s.e.m. **P*<0.05, between indicated groups as calculated by a Mann-Whitney U test. *P* values were corrected for multiple comparisons using a Bonferroni correction.
